# The Intestinal Eukaryotic and Bacterial Biome of Spotted Hyenas: The Impact of Social Status and Age on Diversity and Composition

**DOI:** 10.3389/fcimb.2017.00262

**Published:** 2017-06-16

**Authors:** Emanuel Heitlinger, Susana C. M. Ferreira, Dagmar Thierer, Heribert Hofer, Marion L. East

**Affiliations:** ^1^Research Group Ecology and Evolution of Molecular Parasite Host Interactions, Leibniz Institute for Zoo and Wildlife ResearchBerlin, Germany; ^2^Institute for Biology, Molecular Parasitology, Humboldt UniversityBerlin, Germany; ^3^Department of Evolutionary Ecology, Leibniz Institute for Zoo and Wildlife ResearchBerlin, Germany

**Keywords:** eukaryotome, eukaryome, parasites, amplicon sequencing, spotted hyena, social status, bacterial microbiome, age classes

## Abstract

In mammals, two factors likely to affect the diversity and composition of intestinal bacteria (bacterial microbiome) and eukaryotes (eukaryome) are social status and age. In species in which social status determines access to resources, socially dominant animals maintain better immune processes and health status than subordinates. As high species diversity is an index of ecosystem health, the intestinal biome of healthier, socially dominant animals should be more diverse than those of subordinates. Gradual colonization of the juvenile intestine after birth predicts lower intestinal biome diversity in juveniles than adults. We tested these predictions on the effect of: (1) age (juvenile/adult) and (2) social status (low/high) on bacterial microbiome and eukaryome diversity and composition in the spotted hyena (*Crocuta crocuta*), a highly social, female-dominated carnivore in which social status determines access to resources. We comprehensively screened feces from 35 individually known adult females and 7 juveniles in the Serengeti ecosystem for bacteria and eukaryotes, using a set of 48 different amplicons (4 for bacterial 16S, 44 for eukaryote 18S) in a multi-amplicon sequencing approach. We compared sequence abundances to classical coprological egg or oocyst counts. For all parasite taxa detected in more than six samples, the number of sequence reads significantly predicted the number of eggs or oocysts counted, underscoring the value of an amplicon sequencing approach for quantitative measurements of parasite load. In line with our predictions, our results revealed a significantly less diverse microbiome in juveniles than adults and a significantly higher diversity of eukaryotes in high-ranking than low-ranking animals. We propose that free-ranging wildlife can provide an intriguing model system to assess the adaptive value of intestinal biome diversity for both bacteria and eukaryotes.

## Introduction

Mammalian hosts have a long evolutionary history with the diverse communities of prokaryotes (Dethlefsen et al., 2007; Ley et al., 2008; Douglas and Werren, 2016) and eukaryotes (Hafner and Nadler, 1988; Glendinning et al., 2014), here designated as the intestinal biome, present in their gastrointestinal tract. Although the intestinal biome was traditionally viewed as a rather inert species assembly, recent insights have revealed that its composition is influenced by many host traits and that it in turn can have a considerable impact on its host (Turnbaugh et al., 2007; Graham, 2008; Costello et al., 2012; Wegner Parfrey et al., 2014; Kreisinger et al., 2015). Recently, interest in the community of bacteria in the intestine (often called the microbiome, even though bacteria do not comprise all microscopic organisms in the intestines) has grown (Round and Mazmanian, 2009; Turnbaugh et al., 2009; Lozupone et al., 2012), whereas the communities of unicellular (Wegner Parfrey et al., 2014) and especially multicellular eukaryotes in the gastrointestinal tract (eukaryome or eukaryotome) have received less attention.

Following birth, the mammalian gastrointestinal tract is gradually colonized by prokaryotes and eukaryotes (Palmer et al., 2007; Koenig et al., 2011; Lozupone et al., 2012; Yatsunenko et al., 2012). Throughout an individual's lifespan, the composition of the intestinal biome can be altered by various factors, including diet (Turnbaugh et al., 2009; Amato et al., 2013), as exemplified by the transition from the consumption of milk during infancy (Pond, 1977) to an “adult” diet when weaned. Composition and diversity is also influenced by interactions between species within the intestinal biome (Ezenwa, 2004; Benson et al., 2010; Glendinning et al., 2014; Lee et al., 2014; Kreisinger et al., 2015), developmental changes in immune function (Dowling and Levy, 2014) and age related changes in exposure to prokaryotes and eukaryotes in the environment, food and conspecifics (Palmer et al., 2007; Koenig et al., 2011; Lozupone et al., 2012). Despite these factors that induce variation in the intestinal biome among individuals within a species, there is evidence of species-specific bacterial microbiome signatures in mammals (Ley et al., 2008; Ochman et al., 2010; Yildirim et al., 2010; Degnan et al., 2012; Menke et al., 2014).

While the majority of studies on the diversity and composition of bacterial microbiomes are based on partial sequencing of 16S ribosomal RNA genes, the application of metagenomics (the sequencing of the complete genetic repertoire of the host's intestinal microbiome) has revealed links between the bacterial microbiome of the intestine and the host's metabolism (Gill et al., 2006; The Human Microbiome Consortium, 2012). The bacterial microbiome influences host nutrition, fat storage and the metabolism of vitamins and minerals (Turnbaugh et al., 2009; Tremaroli and Bäckhed, 2012; Leone et al., 2015). Experimental studies with germ-free mice also show that the bacterial microbiome of the intestine can affect postnatal development of the hypothalamic-pituitary-adrenal axis (Sudo et al., 2004). Furthermore, bacterially derived fermentation products from the intestinal microbiome help regulate the maturation of microglia which contribute to an active immune defense of the central nervous system (Erny et al., 2015) and there is now general agreement that the bacterial microbiome influences the host immune system (Round and Mazmanian, 2009; Hooper et al., 2012; Sivan et al., 2015).

The impact of intestinal eukaryotes on their hosts has mostly been studied in relation to the pathologies caused by specific helminths and protozoans (Wegener Parfrey et al., 2011; Andersen et al., 2013; Rajilić-Stojanović and de Vos, 2014). We are not aware of any studies on intestinal biomes comprehensively screening for eukaryotes, including multicellular species. This is surprising, as it has been argued that eukaryotes may have an important ecological function in intestinal ecosystems similar to that of keystone species in terrestrial or aquatic ecosystems (Lukeš et al., 2015). By extension this should particularly apply to large multicellular eukaryotes. Currently, most research on the intestinal biomes of mammals has focused on the bacterial microbiome of humans, wild and captive non-human primates, laboratory mice and zoo animals; hence knowledge on the intestinal biomes of free-ranging wild mammals is limited.

In social mammals, social status often determines access to resources (Clutton-Brock and Huchard, 2013) thereby affecting health status (Sapolsky, 2005), wound healing (Archie et al., 2012), immune gene expression (Tung et al., 2012), immune defenses (Flies et al., 2016; Snyder-Mackler et al., 2016) and the likelihood and impact of pathogen infection (East et al., 2001, 2015; Höner et al., 2012). Social status is also likely to affect the species composition and abundance of bacteria and eukaryotes in the gastrointestinal tract.

We present, to our knowledge, the first study to simultaneously investigate both the bacterial microbiome and eukaryome of a wild mammalian species. We applied a multi-amplicon sequencing approach for bacteria and eukaryotes (metabarcoding) to fresh feces to investigate the effect of social status and age on the (distal) intestinal bacterial microbiome and eukaryome of individually known spotted hyenas (*Crocuta crocuta*) in the Serengeti National Park (NP) in northwestern Tanzania. This highly social carnivore lives in fission-fusion groups termed clans, in which natal females and their offspring are socially dominant over immigrant males (Kruuk, 1972). Migratory movements of ungulates cause large fluctuations in the abundance of food resources in clan territories (Hofer and East, 1993a,b), which profoundly affect the foraging behavior of clan members (Hofer and East, 1993a,b). When migratory ungulates are absent, clan members undertake regular long-distance (approximately 80–140 km) foraging trips (termed commuting trips) to areas containing abundant migratory prey (Hofer and East, 1993b). As access to food within a clan's territory is determined by social status, high-ranking females commute far less often than low-ranking females (Hofer and East, 2003) and their reduced foraging effort is reflected in their lower fecal glucocorticoid metabolite (fGCM) concentrations (Goymann et al., 2001b).

High-ranking females are more often exposed to infectious pathogens than low-ranking females because of their more frequent social interactions with clan members (East et al., 2001). Even so, high-ranking females have lower intestinal parasite burdens than low-ranking females, probably because they can allocate more resources to immune processes than low-ranking females (East et al., 2015). Hence, we expect the bacterial microbiome and the eukaryome of high-ranking animals to be more diverse than those of low-ranking animals, because high species diversity is generally considered an index of ecosystem health (Cardinale et al., 2006; Costello et al., 2012; Reich et al., 2012). In contrast, we expect the intestinal biome of low-ranking animals to be less diverse than those of high-ranking animals.

As in all mammals, the intestinal bacterial microbiome and the eukaryome of juvenile spotted hyenas develops after birth. In our study population, juveniles are more often infected with specific pathogens than adults (Goller et al., 2013; Nikolin et al., 2017), including the eukaryote *Dipylidium* sp. (East et al., 2013). Moreover, juveniles have lower protection from antibodies (East et al., 2001) and their diet includes maternal milk until 12–18 months of age (Hofer and East, 1995). For these reasons we expect the bacterial microbiome and eukaryome of juveniles to be less diverse than that of adults.

We tested our predictions concerning the effects of age and social status by assessing sequence read counts of taxonomically annotated ribosomal sequence variants (RSV) and hence the composition and abundance of genera in the bacterial microbiome and the eukaryome of individually known hosts. We also compared these results with those generated by the classical coprological method of parasite egg or oocyst counts as applied, for instance, by East et al. (2015) to assess whether the results of these methods were strongly correlated.

## Methods

### Study population

The spotted hyena (hereafter hyena) study population included three closely monitored clans that are part of an ongoing long-term research program in the center of Serengeti NP, northwest Tanzania. Individuals were recognized by their spot patterns, ear notches, scars and bald patches (Frank, 1986; Hofer and East, 1993a) and sexed using the dimorphic shape of the phallic gland (Frank et al., 1990). Age was determined from the observed date of birth or based on observations of pelage, position of the ears, level of coordination when walking and body size, with an accuracy of ±7 days as previously described (East et al., 2003). Animals were categorized as juveniles when <24 months of age, and adult when ≥24 months of age. Females were allocated a social rank within the dominance hierarchy using submissive responses in dyadic interactions (East et al., 2003). To compare rank positions across clans, individuals were assigned a standardized rank within a dominance hierarchy by distributing ranks evenly between the highest rank (standardized rank +1) and the lowest rank (standardized rank −1), with the median rank being scored as 0 (Goymann et al., 2001a). Females holding standardized ranks with 0 or above 0 were categorized as high-ranking, those holding a standardized rank of less than 0 as low ranking.

### Sampling

Forty-two fecal samples (35 adult females and 7 juveniles) were collected immediately after defecation from individually known animals between 2009 and 2012. Samples were thoroughly mixed and aliquots were stored in formalin (4%) for parasite egg counts and preserved in RNAlater (Sigma–Aldrich, St Louis, MO, USA) for molecular genetic analyses. Samples in RNAlater were initially stored frozen at −10°C, transported frozen and then stored at −80°C for genetic analysis (East et al., 2013). DNA was extracted using the Macherey-Nagel Nucleo-spin soil DNA extraction kit (Macherey-Nagel, Düren, Germany) following the manufacturer's recommendations and using the Peqlab Precellys 24 homogenisator (VWR International Group, Erlangen, Germany). We assume that fecal biomes are representative of intestinal biomes, since a strong relationship between the two was demonstrated for freshly collected (Menke et al., 2015) and properly stored samples (Menke et al., 2017).

### Parasite egg counts

Parasite egg or oocyst counts were conducted on aliquots of a subset of 32 fecal samples (27 adult females and 5 juveniles, 20 high-ranking and 12 low-ranking individuals) that were suitable for this procedure using a modification of the McMaster flotation technique (Gordon and Whitlock, 1939). To enhance the detectability of eggs, a solution of potassium iodide (KI) was used with a specific weight of 1.5 g ml^−1^ (Meyer-Lucht and Sommer, 2005; Schwensow et al., 2007). Four McMaster chambers were counted for each sample with a dilution factor of 1:15. After combining the feces with the KI solution, it was vortexed for 3 min and then sieved in order to remove bigger debris. Parasite eggs or oocysts were identified according to their morphology and counted using a light microscope, 1 h after preparing the fecal suspension, with a magnification of 100x (10x eyepiece lens × 10x objective lens). Pictures were captured using the software ProgRes CapturePro version 2.5, 2007 (Jenoptik, Jena, Germany). During fecal egg or oocyst counts, eukaryote parasites were identified at the genus or family level on the basis of their morphology and size, with the exception of oocysts from the order Coccidia because they are very similar in terms of their morphological appearance. For *Ancylostoma*, the Taeniidae and the Coccidia two morphological types based on two size classes of eggs or oocysts were distinguished. For the nematode *Ancylostoma*, the two identified size classes for eggs were <80 μm and ≥80 μm, for the cestodes from Taeniidae, the two size classes were <45 μm and ≥45 μm, and for the Coccidia oocysts, the two size classes were <20 μm and ≥20 μm, respectively. The results are expressed as fecal egg counts per g feces (FEC) or fecal oocyst counts per g feces (FOC). All egg and oocyst counts were done blind with respect to the life history stages and characteristics (age, social status) of the individual hyenas from which the fecal sample was taken and analyzed.

### Multi-amplicon PCRs and sequencing

The Fluidigm Access Array integrated fluidic circuit (Fluidigm, San Francisco, California, USA) was used to run 48 × 48 PCR reactions in 2,306 compartments of a microfluidics device. Target specific PCR primers (Supplementary File 1) for 18S and 16S small ribosomal subunits (SSU) were used in a “four primer” PCR approach, following the manual provided by Fluidigm. Briefly, target specific primer pairs were combined with “CS1” and “CS2” adapters at their 5′ and 3′ ends, respectively, on the microfluidics device. These target specific primer pairs were used to prime 48 target specific reactions for each of 48 samples, using default cycling parameters. After harvesting all products from the separate samples into a 96 well microliter plate, a second PCR was performed on a 10-fold dilution, by introducing Illumina sequencing oligonucleotides “PE5” at the “CS1” adapter and “PE7” at the “CS2” adapter as well as a sample identifier sequence between “CS2” and “PE7” based on the Access Array Barcode Library (Fluidigm, San Francisco, California, USA) for Illumina Sequencers (384 single direction). Samples were pooled and selected by size using Agencourt AMPure XP Reagent beads (Beckman Coulter Life Sciences, Krefeld, Germany). For further cleanup, PCR fragments between 400 and 1,000 bp were purified by PippinPrep using the 1.5% agarose DNA gel cassettes (Sage Science Inc., Beverly, Massachusetts, USA). Suitability of PCR products for sequencing (e.g., by checking for an absence of primer multi-meres) was confirmed using the Agilent 2200 Tape Station with D1000 ScreenTapes and D1000 Reagents (Agilent Technologies, Santa Clara, California, USA). Sequences were generated at the Berlin Center for Genomics in Biodiversity Research (BeGenDiv) on the Illumina MiSeq machine (Illumina, San Diego, California, USA) using version 3 chemistry and 600 cycles of (paired-end) sequencing. The sequencing data can be accessed through the accession number PRJNA386767 at NCBI Short Read Archive (SRA).

### Bioinformatic analyses

Sorting of sequencing reads in different samples was performed using the bcl2fastq utility version 2.17.1.14 (Illumina, San Diego, California, USA) based on the sample identifier oligos. All subsequent bioinformatic, taxonomic and statistical analyses were performed in R version 3.3.2 (R Development Core Team, 2016); below we cite the R packages used for specific steps in the analysis.

Sequences were quality trimmed and screened for erroneous reads using the fastqPairedFilter function of package dada2 version 1.2.1 (Callahan et al., 2016) with parameter settings of truncLen = c(170,170), maxN = 0, maxEE = 2, truncQ = 2. Further stratification of the full “samples by amplicon” matrix was performed using package MultiAmplicon version 0.1 (Heitlinger, 2017): primer sequences were trimmed in read pairs matching with zero mismatches starting at position one in both forward and reverse reads. Sequencing reads were sorted into an amplicon when they contained the sequences of a specific pair of primers. The MultiAmplicon package was also used as a wrapper to process identified amplicons with the dada2 workflow. Briefly, sequences were dereplicated, RSVs were inferred using the function dadaMulti (with options err = NULL, selfConsist = TRUE), forward and reverse reads were concatenated (using function mergeMulti, option justConcatenate = TRUE), a table of RSV occurrence was collated for each sample and sequences likely to be chimeric and introduced during PCR were screened and discarded.

Technical replicates for which PCRs failed were identified by hierarchical clustering of primer-stratified read numbers, with water as negative controls, and marked for exclusion. For each sample, RSV counts were obtained as the sum of the remaining technical replicates and normalized for sequencing depth using a simple scaling by the median of the per sample RSV counts. All taxonomic assignments were done blind with respect to the life history stages and characteristics (age, social status) of the individual hyenas from which the fecal sample was taken and analyzed.

### Taxonomic and statistical analyses

Taxonomy was inferred for RSVs using a ribosomal database project naïve Bayesian classifier (Wang et al., 2007) through dada2's “assignTaxonomy” function. As a training data set for the classifier, the SILVA_123 database (Quast et al., 2013) was expanded with highest scoring unique BLAST hits (blastn; Altschul et al., 1997) of our RSVs in the NCBI nt database. This training dataset was thus curated with a focus on eukaryotic 18S sequences in our study system and then used in an assignment of the taxonomy levels “phylum,” “class,” “order,” “family,” and “genus” to each of our RSVs. Problems arose for annotation of putative *Eimeria* reads (among others) because of an incongruence between the taxonomic systems of SILVA and NCBI, so two separate sets of results will be described for this genus.

RSV counts, annotation with taxonomy information and hyena specific sample data were combined into a single R object for all amplicons using the package phyloseq version 1.18.0 (McMurdie and Holmes, 2013). Data were merged across different amplicons via their taxonomic annotation at the genus level using the function “tax_glom” with the parameter “NArm = TRUE.” This excludes genera not annotated at the genus level (or annotated with uninformative terms such as “undefined”). We also excluded genera annotated as the only genus in their respective phylum and genera with an “undefined” phylum annotation. We report estimates of RSV diversity derived from different amplicons with caution, as we recognize that many raw RSVs prior to taxonomic annotation represent different parts of the same marker genes and hence our diversity estimates are inflated for this measure. Even so, they can be used to compare the diversity between individuals, because all samples were processed using the same amplification and bioinformatics workflow.

We used the strength of association between “sequence abundance” (number of ribosomal sequence reads annotated for specific genera or higher level taxa) and FEC or FOC to screen for the most likely sequenced taxon in samples for which we had morphological evidence from the egg or oocyst counts. Because the larger size class of *Ancylostoma* occurred in only two samples, FEC for both size classes were added together for the correlation of egg counts with sequence abundance data. Spearman rank correlation coefficients were calculated in R base and recorded not only for “target taxa” within the taxonomic scope of morphological discrimination but in a “blinded” approach for the comprehensive set of all sequence counts at all levels of taxonomy. The strongest correlations were then screened for taxonomic agreement with FEC and FOC. The four highest positive correlations always contained the target taxa as defined by the observed morphology. In the results (below) we report *p*-values to test for significant association between the number of ribosomal sequence reads and FEC or FOC for the highest or second highest Spearman correlation coefficients. We also constructed linear models using (1+log_10_) transformed data to visualize the linearity of the association and report the regression equations as a predictive tool.

Richness—the number of taxa (RSVs or genera) present —, evenness—the evenness in the distribution of sequencing read numbers for different taxa—and diversity—an index that takes into account both richness and evenness—(see Legendre and Legendre, 2012) were calculated after the random selection of a data subset (rarefaction) from all sample counts at the sequencing depth of the library sequenced to the lowest depth. We used rarefaction instead of normalization for diversity estimates to avoid problems of overestimating presence in more deeply sequenced samples. We used the package phyloseq with its function estimate_diversity to estimate measures of diversity and species richness with the help of package vegan version 2.4-1. We calculated the number of genera as “observed richness,” the Chao1 index of diversity (Chao, 1984) as a measure of species diversity and Pielou's *J* as a measure of evenness (Pielou, 1975). Number of genera, Chao1 index and Pielou's *J* were compared between juveniles and adults or high-ranking and low-ranking hyenas using the exact version of the Mann-Whitney U test in the package coin version 1.1-2 (Hothorn et al., 2006) to obtain appropriate *p*-values for small sample sizes and in the presence of ties.

In order to compare the composition of the bacterial microbiome and the eukaryome between individuals, life history stages and social status categories, the diversity of the bacterial microbiomes and eukaryomes and its underlying variation across individuals was efficiently summarized by multidimensional scaling (MDS), a non-parametric ordination technique. This technique has previously been highly successful in summarizing similarly complex data sets (e.g., Burgener et al., 2009), along new dimensions called here MDS axes. MDS uses pairwise Bray-Curtis dissimilarities (Bray and Curtis, 1957) on (1+log_10_) transformed data of sequence abundances per genus. On the same transformed data partial least squares (PLS) models (Hastie et al., 2013), as calculated by package caret version 6.0-73, were used as a supervised machine learning technique (Wold et al., 1984) to predict age and rank category for the individual from which the sample was collected. PLS models also produce a set of axes or “directions” (Hastie et al., 2013) called here PLS axes. The optimal number of PLS axes retained in the final model was determined using leave-one-out cross-validation. Each sample received a PLS score on each PLS axis, which documents how well sample categories can be differentiated on that axis, and each taxonomic unit as a “predictor” received a PLS “loading” on each axis which documents to what extent the taxonomic unit contributed to the PLS axis. We subsequently used Fisher's exact test from R base to test for overrepresentation or underrepresentation of phyla visually identified to be abundant at the extremes of the distribution of loadings. We tested highest and lowest quartile of loadings on the single PLS axis of the model addressing rank category differences in the eukaryome. As the PLS model for differences of the microbiome in age categories contained more than one axis, the PLS loadings of the first two axes separating the samples were combined by multiplication before this procedure.

Package DESeq2 version 1.14.0 (Love et al., 2014) (function “DESeq”) was used to test for differences in the abundance of individual genera between age and rank categories. In contrast to normalization or rarefaction used in other analyses the function estimated “size factors” to address differences in sequencing depth between different samples. These factors were then used as offset when a mean abundance was fitted for each taxon in generalized linear models (glm) using a negative binomial distribution and a dispersion parameter specific to that taxon. Maximum likelihood estimates for glm coefficients were obtained and likelihood ratio tests were conducted by subtracting the log-likelihood of the full model including different estimates for a focal contrast (age or rank category) from a reduced model without this difference. The resulting likelihood-ratio was compared then to a χ^2^-distribution. Resulting *p*-values were corrected for multiple testing using the Benjamini and Hochberg method (Benjamini and Hochberg, 1995) and expressed as false discovery rates, with the significance threshold set to 0.05.

## Results

High throughput sequencing of multiple amplicons provided a comprehensive survey of the intestinal biome of hyenas, comprising 986 genera, most of which belonged to the eukaryotic biome, as described in detail below.

### Sequencing based assessment

We obtained a total of 3,195,831 sequencing read-pairs from amplicon sequencing for 18S small ribosomal subunits of eukaryotes and 16S ribosomal subunits of bacteria. Variant inference on a single base resolution level for these sequences revealed a total of 24,604 RSVs, which were summarized via shared taxonomic assignments at the genus level.

### Taxonomic diversity of hyena intestinal biomes

**Figure 1:**
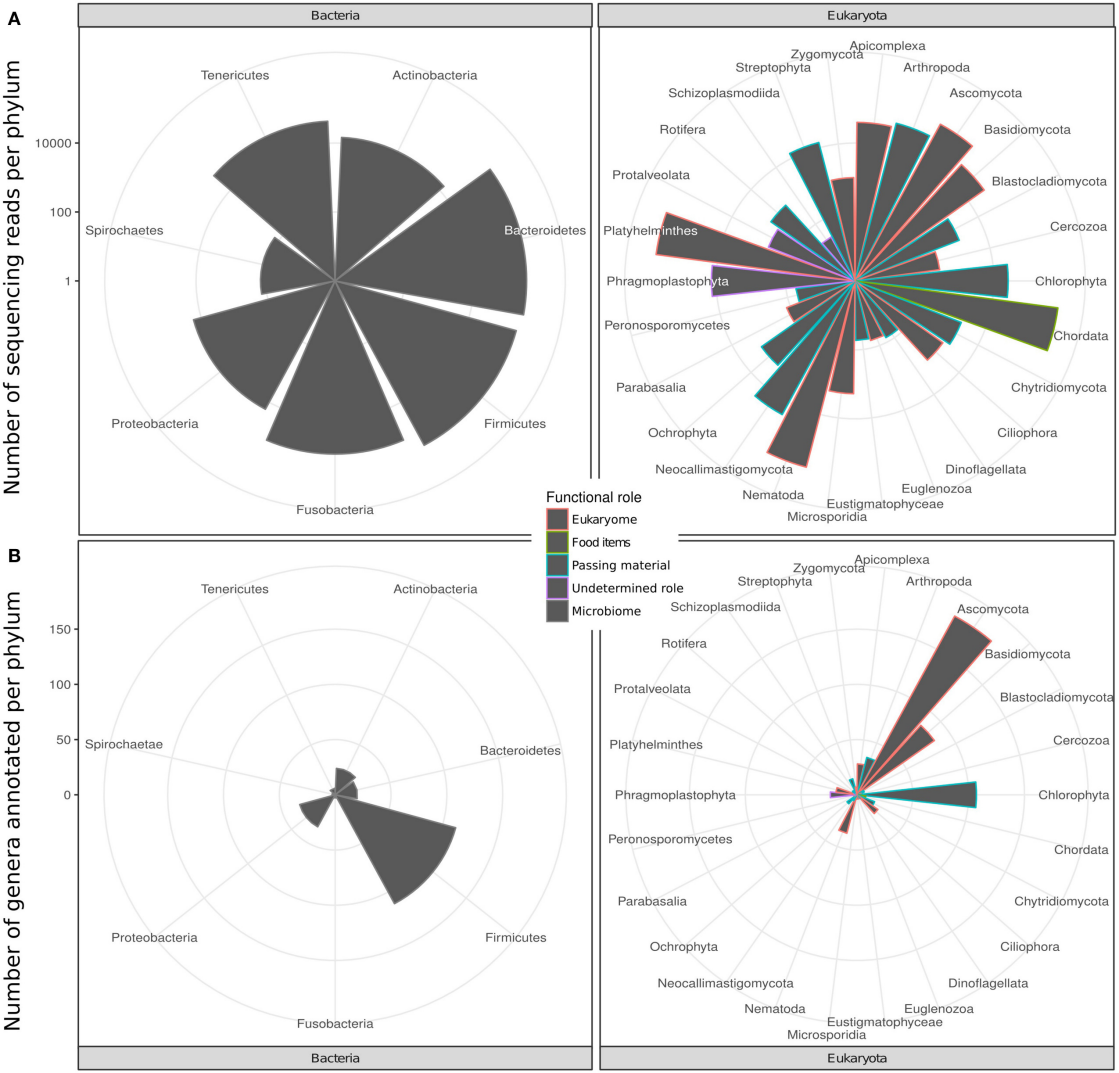
Taxonomic diversity of the intestinal bacterial microbiome and eukaryome of the spotted hyena in terms of **(A)** number of genera and **(B)** number of sequence reads inferred for different phyla of bacteria and eukaryotes from taxonomically annotated multi-amplicon sequencing reads. Bars are colored according to the likely predominant interaction with their host conventionally assumed for these taxa (see main text).

We identified 201 genera (3,725 RSVs) of bacteria as constituents of the bacterial microbiome of the hyena. We also identified 656 genera (20,879 RSVs) of eukaryotes in the same fecal samples. The number of genera annotated varied greatly between phyla of both bacteria and eukaryotes (Figure 1A, Table 1). High genus level diversity was observed in the bacterial phylum Firmicutes and in the eukaryote phyla Ascomycota, Chlorophyta and Basidiomycota. The classic phyla of intestinal parasites—Nematoda, Apicomplexa and Platyhelminthes—showed an intermediate number of genera but a high number of sequencing reads. Phylum Chordata was represented with few genera and a large number of sequencing reads (Figure 1A, Table 1).

**Table 1:** The diversity of genera and phyla of bacteria and eukaryotes recorded from the intestinal biome of the spotted hyena as extracted by amplicon sequencing of fecal samples.

**Taxon**	**Functional role**	**Ribosomal sequencing variants (RSV)**	**Identified genera**	**Number of sequencing reads**
**Bacteria**	**Microbiome**	3,559/^*^3,725	201/^*^202	768,581/^*^807,654
Actinobacteria	Microbiome	184	24	14,690
Bacteroidetes	Microbiome	787	20	328,848
Firmicutes	Microbiome	2,073	112	259,513
Fusobacteria	Microbiome	183	3	105,138
Proteobacteria	Microbiome	248	33	16,881
Spirochaetes	Microbiome	13	3	140
Tenericutes	Microbiome	71	6	43,371
Undetermined^**^	Microbiome	166	1^**^	38,928
**Eukaryotes**	**Multiple**	18,202/^*^20,879	656/^*^784	2,129,418/^*^2,388,177
Apicomplexa	Eukaryome (parasites)	1,074	28	39,899
Arthropoda	Food items	600	35	53,075
Ascomycota	Eukaryome	4,841	184	146,864
Basidiomycota	Eukaryome	1,675	85	36,241
Blastocladiomycota	Passing material	126	7	1,676
Cercozoa	Eukaryome	47	4	288
Chlorophyta	Passing material	1,131	107	25,680
Chordata	Food items	1,743	8	782,156
Chytridiomycota	Passing material	144	17	1,944
Ciliophora	Eukaryome	254	32	1,264
Dinoflagellata	Passing material	30	4	74
Euglenozoa	Eukaryome	13	4	61
Eustigmatophyceae	Passing material	6	3	53
Microsporidia	Eukaryome (parasites)	39	4	1,869
Nematoda	Eukaryome (parasites)	1,431	36	380,347
Neocallimastigomycota	Passing material	337	6	25,520
Ochrophyta	Passing material	184	11	2,008
Parabasalia	Eukaryome	29	3	131
Peronosporomycetes	Passing material	11	3	52
Phragmoplastophyta	Undetermined role	420	24	13,602
Platyhelminthes	Eukaryome (parasites)	1,195	19	600,119
Protalveolata	Undetermined role	85	3	478
Rotifera	Passing material	100	5	930
Schizoplasmodiida	Undetermined role	11	2	28
Streptophyta	Passing material	107	15	14,066
Zygomycota	Eukaryome	98	7	993
Undetermined^**^	Undetermined role	2,471	128^**^	216,753

* *The first number is for the annotated dataset analyzed for genera; the second number is the full dataset analyzed for RSVs*.

** *Number of genera not considered correctly annotated based on phylum level abundance (see methods) or annotated as “undefined” at the phylum level*.

The eukaryote sequences derived from 18S amplification did not solely originate from organisms in the eukaryome but also included organisms that were part of the hyena's diet or those that were accidentally ingested. Taxonomic annotation of eukaryote derived DNA can help to assess these potential sources. For some taxa a role as intestinal inhabitants or food items can safely be assigned. We therefore categorized eukaryote phyla in our taxonomic annotation in categories of likely (1) eukaryome (including parasites, commensals and mutualists), (2) food items, (3) passing material, and (4) undetermined role (Table 1). We consider for example Nematoda, Platyhelminthes, Apicomplexa and Microsporidia as classical eukaryotic parasites and hence members of the eukaryome. Other organisms not conventionally considered to be parasites but rather commensals or with an unknown effect on their host comprised most phyla of fungi. DNA sequences from the phylum Chordata were most likely originating from hyena food items (Table 1).

### Sequence based abundance counts correlate with fecal egg or oocyst counts but are more sensitive

Eukaryote parasites were identified at a genus level for the genus *Ancylostoma* (detected in 25 of 32 samples), more conservatively at the family level for cestodes in families Diphyllobothriidae (detected in 26 of 32 samples) and Taeniidae (detected in 6 of 32 samples) and protists only at the level of order for Coccidia (detected in 17 of 32 samples). We also detected in one or two samples species from Spirurida, *Trichuris* spp. and *Dipylidium* spp. (Table 2).

**Table 2:** The intensity of infection in terms of parasite egg or oocyst counts per g feces from fecal samples (*n* = 32) of spotted hyenas.

**Taxon**	**Taxon level**	**Phylum**	**Prevalence**	**Intensity of infection**					
			**%**	**Mean**	**Confidence intervals**		**Median**	**Confidence intervals**	
					**Low**	**High**		**Low**	**High**
Diphyllobothriidae	family	Platyhelminthes	81.2	21,461	1,137	41,784	3,650	850	6,850
*Ancylostoma* total	genus	Nematoda	78.1	802	320	1,284	275	100	773
*Ancylostoma* < 80 μm	spp.	Nematoda	78.1	786	308	1,264	275	100	773
*Ancylostoma* ≥ 80 μm	spp.	Nematoda	6.2	200	0 [−94]	494	200	NA	NA
Coccidia total	order	Apicomplexa	53.2	2,171	0 [−664]	5,007	50	25	75
Coccidia < 20 μm	spp.	Apicomplexa	12.5	8,800	0 [−1504]	19,104	6,588	NA	NA
Coccidia ≥ 20 μm	spp.	Apicomplexa	53.1	101	29	173	50	25	50
Taeniidae total	family	Platyhelminthes	18.8	89	15	164	62	25	75
Taeniidae < 45 μm	spp.	Platyhelminthes	3.1	85	10	161	50	25	75
Taeniidae ≥ 45 μm	spp.	Platyhelminthes	18.8	25	NA	NA	25	NA	NA
Spirurida	order	Nematoda	6.2	412	0 [−249]	1,074	412	NA	NA
*Trichuris*	genus	Nematoda	3.1	50	NA	NA	50	NA	NA
*Dipylidium*	genus	Platyhelminthes	3.1	50	NA	NA	50	NA	NA

*Indicated are identified taxa, their prevalence in %, the mean and median with respective 95% confidence intervals. Table was sorted by prevalence. “spp.”: an unknown number of species which may or may not belong to more than one genus*.

**Figure 2:**
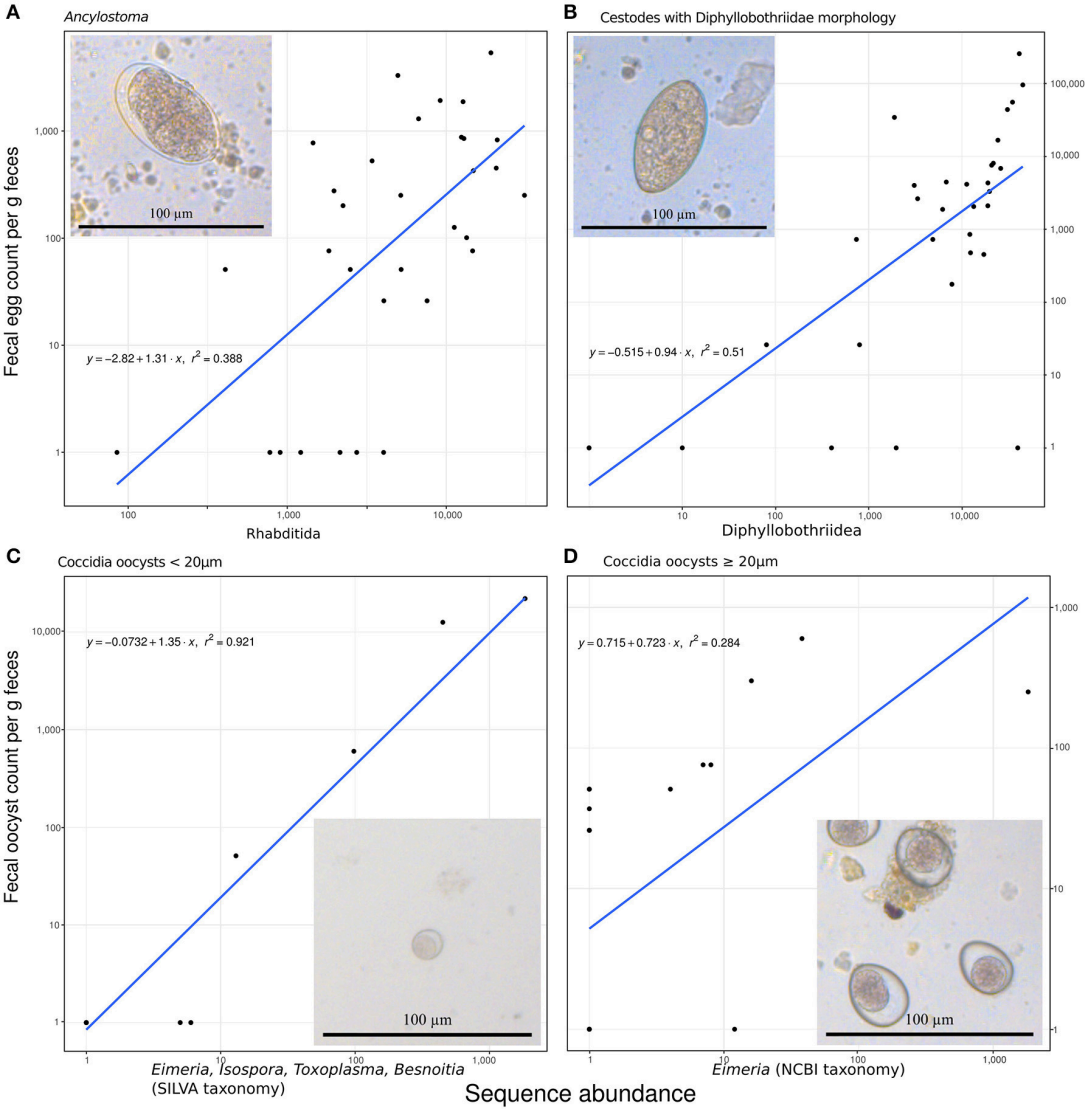
Predicting fecal egg or oocyst counts per g feces from the number of annotated ribosomal sequence variants reads. **(A)** *Ancylostoma* FEC vs. sequence counts for the order Rhabditida. **(B)** Diphyllobothriidae FEC vs. sequence counts for the same family. **(C)** A small size class of oocyst counts vs. added sequence counts for *Eimeria, Isospora, Besnoitia*, and *Toxoplasma*. **(D)** A large size class of coccidian oocysts vs. *Eimeria* sequences. All panels contain the formula for the specific linear model on (1+log_10_) transformed data, R^2^ as a measure of goodness of fit, and a line representing the predicted relationship. The panels additionally include a representative micrograph depicting the egg or oocyst counted.

The amplicon sequencing based abundance estimates correlated significantly and positively with egg or oocyst counts, as detailed below, for those species for which we had more than six positive FEC or FOC samples. These correlations also helped to assign plausible taxonomic status to taxa with morphologically indistinguishable eggs or oocysts. *Ancylostoma* FEC correlated best with the sequence counts for the genus *Ancylostoma* among all reported genera (Spearman's rho, ρ = 0.54, *n* = 32, *p* = 0.002). There was a slightly better positive correlation with counts summarized for the order Rhabditida, to which *Ancylostoma* belongs (ρ = 0.58, *n* = 32, *p* < 0.001): seven samples with relatively high sequencing counts (range 84–4,035) and zero FECs were recovered and all samples reporting FEC in Rhabditida had at least 407 sequences counted for *Ancylostoma*. Within this order, annotations for 23 other genera were reported. The genus *Ancylostoma* contributed most (59%) sequencing reads annotated within the order. The genera *Ostertagia* (20%) and *Haemonchus* (12%) also contributed substantial numbers of sequencing reads annotated as Rhabditida. Adding together reads annotated as *Ancylostoma* and *Haemonchus* resulted in a correlation slightly stronger (ρ = 0.59, *n* = 32, *p* < 0.001) than that observed for the whole order Rhabditida. When reads classified as *Ostertagia* were added to the reads annotated as *Ancylostoma*, the correlation with *Ancylostoma* spp. in the FEC became slightly weaker (ρ = 0.54, *n* = 32, *p* = 0.002) (Figure 2A).

Similarly, FEC for taxa identified as originating from one or several species in the family Diphyllobothriidae correlated best with annotated RSV counts for the family Diphyllobothriidae (ρ = 0.69, *n* = 32, *p* < 0.001). The genus *Diphyllobothrium* provided the vast majority (98%) of counts annotated in the family Diphyllobothriidae and reads for the genus *Spirometra* provided the remaining (2%) of counts in this family. The correlation of FEC with annotated RSV counts for *Spirometra* alone was slightly weaker (ρ = 0.67, *n* = 32, *p* < 0.001) than for the entire family. The FEC results for four samples contained no *Diphyllobothrium* eggs but produced positive *Diphyllobothrium* sequence results, with annotated RSV counts ranging from 9 to 39,699; all samples with FEC above zero produced annotated RSV counts in the range of 79–44,993 for Diphyllobothriidae. Figure 2B visualizes this relationship.

When annotated RSV counts for the genera *Besnoitia, Toxoplasma, Isospora*, and *Eimeria* were added together, their total number correlated best (ρ = 0.79, *n* = 32, *p* < 0.001) with FOC for the small size class (<20 μm) of Coccidia oocysts. This comparison produced a linear model on log transformed data with an excellent fit (*r*^2^ = 0.92; Figure 2C). FOC were zero for three samples with sequencing counts ranging from four to five for these genera. The number of RSV counts annotated as *Eimeria* correlated best (ρ = 0.60, *n* = 32, *p* < 0.001) with the large size class (≥20 μm) of Coccidia oocyst counts (Figure 2D). This relationship did not follow the pattern reported for other correlations, since substantial numbers of FOC were reported for samples with zero abundance in terms of annotated RSV reads.

### Adult female hyenas have a bacterial microbiome which is more diverse than and differs in composition from that of juveniles

**Figure 3:**
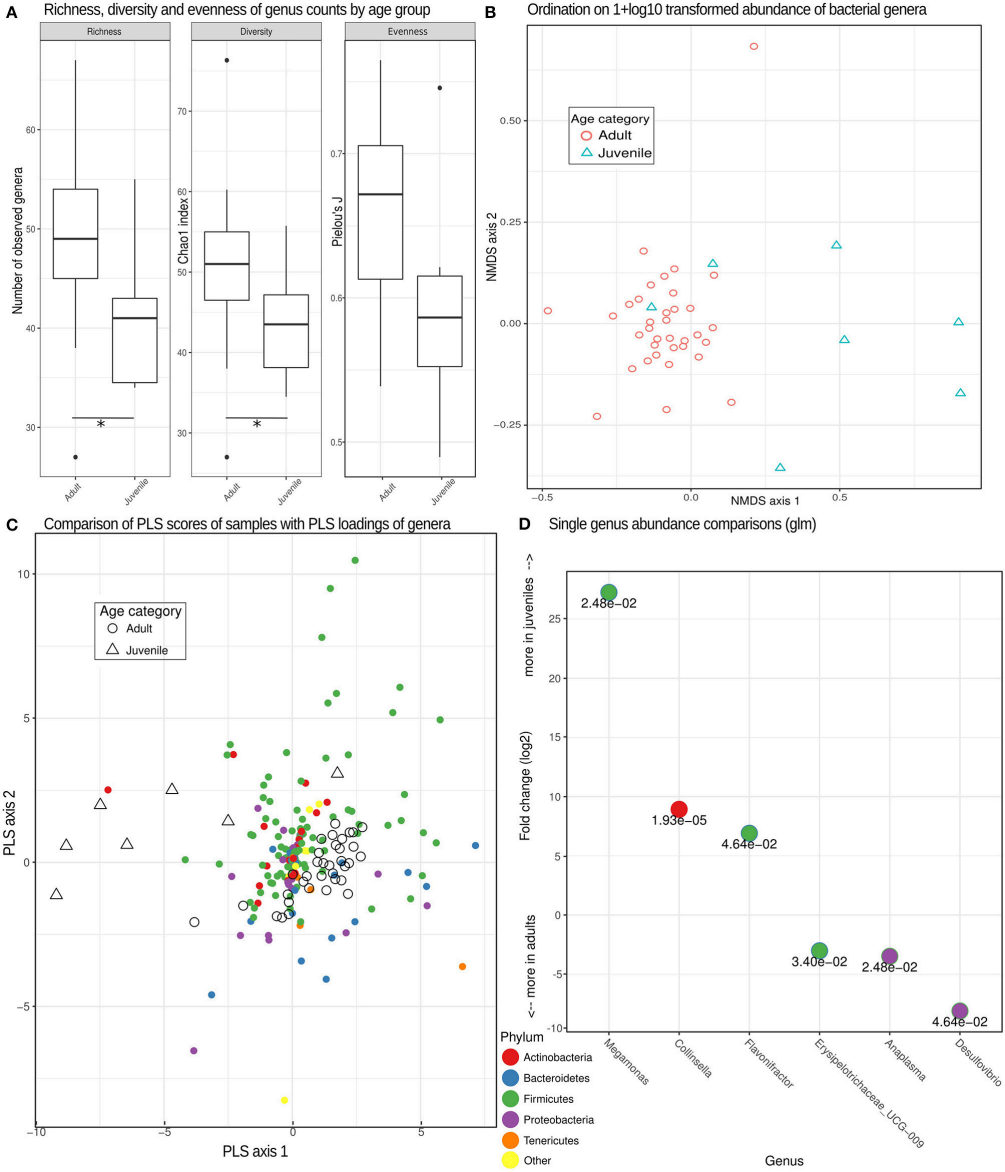
Bacterial genera richness, diversity and microbiome composition in different age categories. **(A)** Box plots depicting distributions of richness (observed counts of genera richness per phylum), diversity (Chao1 index) and evenness (Pielou's *J*) estimates on rarefied (see methods) genera counts for juveniles and adults. ^*^Significant differences (*p* < 0.05) based on exact Mann-Whitney U tests. **(B)** Non-metric multidimensional scaling (MDS) ordination based on pairwise Bray-Curtis dissimilarities partially separated juvenile from adult samples based on different compositions of taxonomic units. **(C)** A comparison of PLS scores (for samples) and PLS loadings (for genera) from the first two PLS axes of an optimized partial least squares model, demonstrating a clear separation of adult and juvenile samples. Genera colored by phylum can be used to assess the taxa contributing (PLS loading) to the differences underlying this distinction. **(D)** Log2-fold change inferred by generalized linear models testing for differences between adults and juveniles for each genus with a false discovery rate (adjusted *p*-value) of < 0.05. The numerical value of the false discovery rate is given below the dot for each genus color-coded for its respective phylum.

Consistent with our prediction, the bacterial microbiome of juvenile hyenas contained a significantly lower number of genera (lower richness; Mann-Whitney U-test, *U* = 184.5, *n* = 42, *p* = 0.012), had a lower diversity (*U* = 174.5, *n* = 42, *p* = 0.034) and showed a trend toward lower evenness (*U* = 168, *n* = 42, *p* = 0.063) than that of adults (Figure 3A). The bacterial microbiome of adult hyenas contained a median of 49 bacterial genera whereas those of juveniles hosted a median of 41 genera.

A more detailed analysis for each bacterial phylum revealed that the higher richness and diversity of genera in adults than juveniles predominantly occurred in the phyla Tenericutes (*U* = 203, *n* = 42, *p* < 0.001) and Bacteroidetes (*U* = 173, *n* = 42, *p* = 0.037). Microbiomes of adults had a significantly lower diversity for Actinobacteria than those of juveniles (*U* = 53, *n* = 42, *p* = 0.021). The differences in composition between the bacterial microbiomes of juveniles and adults were confirmed using multidimensional scaling ordination, which showed that juvenile microbiomes are less uniform in composition between individuals than those of adults (Figure 3B).

The distinct composition of adult and juvenile bacterial microbiomes was underlined by the results of a PLS regression which correctly assigned samples to age categories with an accuracy of 93% in leave-one-out cross evaluations. The optimal model retained three PLS axes, the first two of which are plotted with PLS scores of samples and loadings of bacteria phyla in Figure 3C, illustrating how well age categories were separated by their PLS scores. A more detailed analysis demonstrated that genera in the phyla Tenericutes tended to be (odds ratio = 4.83, Fisher test, *p* = 0.069) and Bacteroidetes were (odds ratio = 5.42, Fisher test, *p* < 0.001) characteristic for adult microbiomes, Actinobacteria for juvenile microbiomes (odds ratio = 2.93, Fisher test, *p* = 0.009). Testing individual genera of bacteria for differences in abundance between age categories resulted in six genera with significant false discovery rates of <0.05, with three more abundant in juveniles and three more abundant in adults (Figure 3D).

In contrast to the bacterial microbiome, we detected no significant differences in eukaryome richness (*U* = 106, *n* = 42, *p* = 0.75), diversity (*U* = 96, *n* = 42, *p* = 0.50), evenness (*U* = 128, *n* = 42, *p* = 0.68) or genus abundance between hyena age categories (false discovery rate for all single genera glms >0.05).

### A more diverse eukaryome in high-ranking than low-ranking hyenas

**Figure 4:**
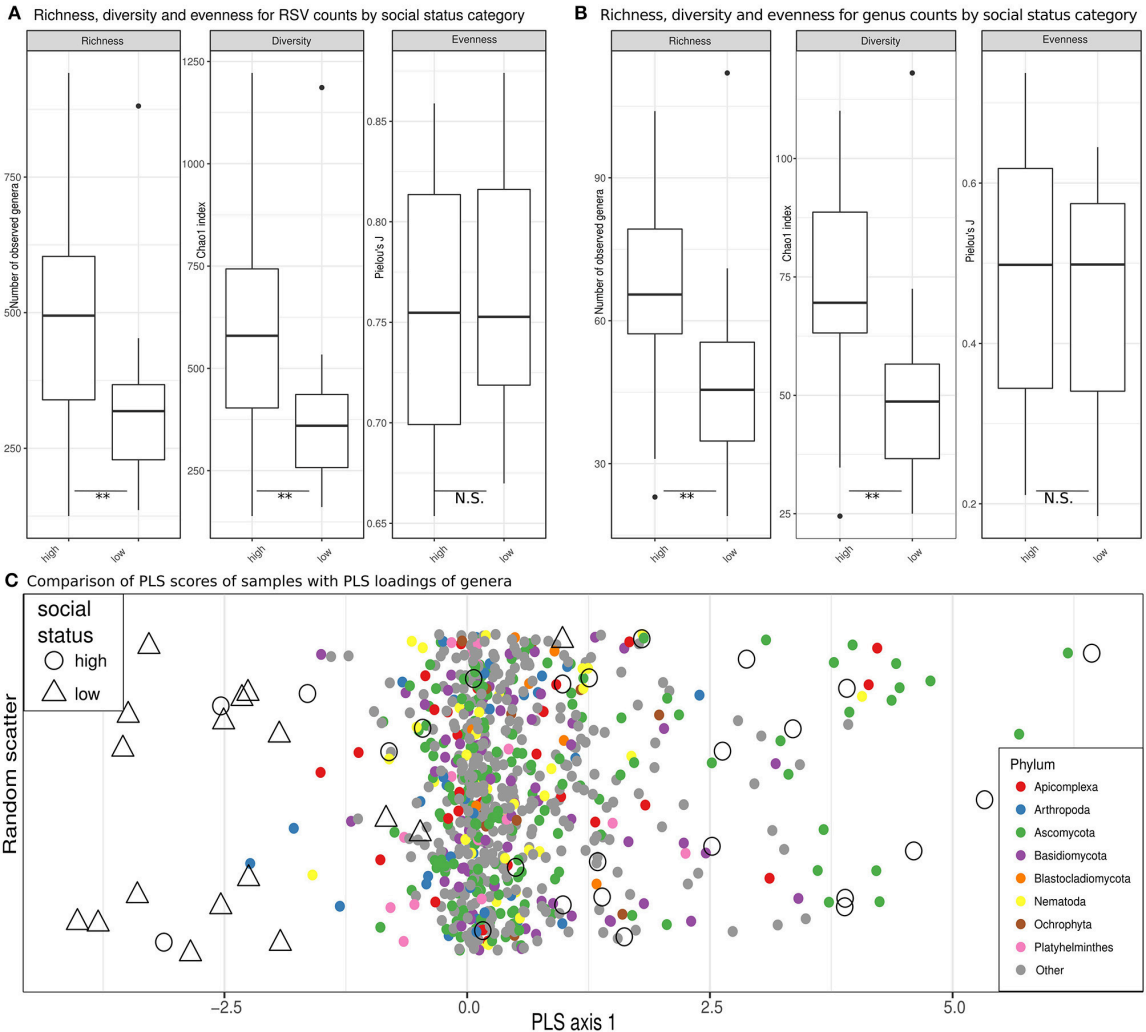
Eukaryome diversity and composition in high ranking vs. low ranking hyenas. **(A)** Estimates of richness (observed counts of genera richness per phylum), diversity (Chao1 index) and evenness (Pielou's *J*) on rarefied (see methods) ribosomal sequence variant (RSV) counts for high-ranking and low-ranking individuals. **(B)** Counts for annotated RSV per genus are compared in box plots for high and low ranking hyenas. ^**^Significant differences (*p* < 0.01) based on exact Mann-Whitney U tests. **(C)** A comparison of PLS scores (for samples) and PLS loadings (for genera) are visualized on the single PLS axis of an optimized partial least squares model, demonstrating a separation of the majority of samples from high-ranking individuals from samples from low-ranking animals. On the y-axis random scatter is introduced for visualization. The underlying genera are color-coded for their respective phylum.

Estimates of observed richness (*U* = 293, *n* = 42, *p* = 0.004) and diversity (U = 288, *n* = 42, *p* = 0.007) in terms of RSVs in the eukaryome were significantly higher in high-ranking than low-ranking hyenas (Figure 4A). The same was true for inferred genera (richness, *U* = 299, *n* = 42, *p* = 0.002; diversity, *U* = 299.5, *n* = 42, *p* = 0.002; Figure 4B). Social status had no significant effect on eukaryome evenness. A significantly higher number of genera (richness) occurred in high-ranking than low-ranking individuals for the phyla Basidiomycota (*U* = 275, *n* = 42, *p* = 0.021), Ascomycota (*U* = 272.5, *n* = 42, *p* = 0.025) and Blastocladiomycota (*U* = 250, *n* = 42, *p* = 0.049). There was a trend toward higher diversity in high-ranking than low-ranking hyenas for Apicomplexa (*U* = 254.5, *n* = 42, *p* = 0.079).

It was not possible to determine compositional differences between eukaryomes of high-ranking and low-ranking animals in our dataset using unsupervised ordination techniques. PLS regression models, however, were able to assign animals to social status categories with 71% accuracy in leave-one-out cross evaluations. Most high-ranking individuals were clearly separated from low-ranking individuals by the single PLS axis retained in the model, the overlap with low-ranking individuals being confined to a minority of high-ranking individuals (Figure 4C). The loading of several genera belonging to the phylum Basidiomycota tended to increase the PLS scores for high-ranking individuals (odds ratio = 1.55, Fisher test, *p* = 0.074). There was also a trend that genera in the Apicomplexa through their loadings increased the PLS scores for low-ranking individuals (odds ratio = 2.01, Fisher test, *p* = 0.057). Both trends contributed to the separation of high-ranking and low-ranking individuals. Interestingly, different genera in the Apicomplexa were drivers in terms of their PLS loadings on the single PLS axis for the classification of samples as belonging to both high-ranking and low-ranking individuals (odds ratio = 3.17, *p* = 0.011; testing the combined upper and lower quartiles of loadings).

In contrast to the eukaryome, we detected no significant differences in bacterial microbiome richness (*U* = 184.5 *n* = 42, *p* = 0.84), diversity (*U* = 182.5, *n* = 42, *p* = 0.80), evenness (*U* = 184, *n* = 42, *p* = 0.84) or genus abundance between high-ranking and low-ranking animals (false discovery rate for all individual genera glms >0.05).

## Discussion

Amplicon sequencing (metabarcoding) revealed that spotted hyenas in the Serengeti NP had a diverse intestinal biome, with at least 201 identified genera of bacteria and 656 identified genera of eukaryotes (Table 1, Figure 1A). The bacterial phylum with the highest diversity of identified genera was the Firmicutes, as were the Ascomycota, Chlorophyta and Basidiomycota among the eukaryotes (Figure 1B). Positive, significant correlations between amplicon sequence abundance estimates and FEC or FOC (Figure 2) suggest that results from amplicon sequencing are quantitatively valid and highly sensitive measures, and also can aid the differentiation of taxa with similar egg and oocyst morphologies. Consistent with our predictions, adult females had a significantly more diverse intestinal bacterial microbiome than juveniles (Figure 3) and the diversity of eukaryotes was significantly greater in high-ranking than low-ranking animals (Figure 4). However, contrary to our prediction, there was no effect of social status on the diversity of the bacterial microbiome and also no difference between the diversity of eukaryotes in adult and juvenile intestinal biomes.

Studies of the intestinal bacterial microbiome in humans report an increase in the number of taxa and taxonomic diversity of bacteria with age (Palmer et al., 2007; Tannock, 2007; Koenig et al., 2011). Our results were similar in that the distal intestinal bacterial microbiome of adult hyenas was significantly more diverse and differed in composition from that in juvenile hyenas. Probably both social and physiological factors contributed to these age related differences. During approximately the first 12 months of life, juveniles remain at the clan's communal den (Hofer and East, 1993c) and for most daylight hours they rest together in underground burrows where conditions are probably conducive to the spread of bacteria among resting juveniles (Höner et al., 2012). Dens also function as important social centers where clan members meet and interact, thereby enhancing the transmission of hyena-associated pathogens between clan members (East et al., 2013; Olarte-Castillo et al., 2016). Greeting ceremonies (East et al., 1993), in which participants stand head-to-tail and sniff and lick each other's anogenital area, are probably important for the spread of bacteria and their inclusion in the intestinal biome by the fecal-oral route. Since juveniles frequently participate in greeting ceremonies (East et al., 1993), greetings probably aid the colonization of the juvenile intestinal biome by both bacteria and eukaryotes. In line with this idea there is growing evidence that socially mediated transmission contributes to the maintenance of a diverse bacterial microbiome in a taxonomically broad range of species, including chimpanzees (*Pan troglodytes*), in which social processes help maintain a diversity rich in commensals and mutualists (Moeller et al., 2016), and social insects where social transmission of bacteria provides protection against virulent pathogens (Koch and Schmid-Hempel, 2011). Throughout the long period in which they are nursed, juvenile hyenas receive bacteria from their mother, and the nutritional and immunological components of milk (Hofer et al., 2016) may also affect the composition of the intestinal bacterial microbiome of juvenile hyenas.

As high-ranking individuals are highly sought after social partners, we predicted a socially mediated spread of a more diverse bacterial microbiome in high-ranking than low-ranking individuals but our results did not show this. One possible explanation is that our resolution, to the level of RSVs and genera, was insufficient. Our approach might have failed to distinguish functional differences in the relationship (e.g., mutualistic vs. pathogenic) of variants of the same species with their hosts, as in the case of distinct variants of *Escherichia coli* (von Mentzer et al., 2014). Hence our taxonomic resolution—even at the RSV level—was possibly insufficient for a fine-grained analysis of the diversity of the bacterial microbiome and the functional relationships of variants to their host. An alternative interpretation of our results is that high rates of contact between adult female clan members homogenize their intestinal bacterial biomes, as proposed by ecological network theory (Wilson, 1992).

In contrast to the bacterial microbiome, intestinal eukaryotes are—with the exception of some fungi—traditionally considered parasites and thus detrimental to their host. However, extending the findings made on bacteria in the last decades it seems likely that the mammalian intestinal biome also includes diverse commensals and mutualists (Wegner Parfrey et al., 2014; Lukeš et al., 2015). In microbial communities at least, an increase in species richness increases the potential for metabolic interactions and dependencies between community members, resulting in more stable communities because they become more independent from the environment (Zelezniak et al., 2015). More stable communities are less likely to suffer perturbations in their composition (dysbiosis) as reported for many diseases. To what extent this also applies to intestinal eukaryotic communities is unclear at present. We found that high-ranking animals had a significantly more diverse eukaryome than low-ranking animals and we interpret this result to indicate a healthier intestinal ecosystem in high-ranking animals. Previously we have shown that female social status determines access to food resources within the clan territory and foraging effort (Hofer and East, 1993b). As a result, low-ranking females have higher foraging costs and higher fGCM concentrations (Goymann et al., 2001b) indicative of an elevated allostatic load. Low-ranking adult females more often resort to resource allocation trade-offs that reduce allocation of resources to immune processes, which therefore resulted in higher burdens of the intestinal helminth *Ancylostoma* spp., especially during lactation (East et al., 2015). A greater allocation of resources by high-ranking females to immune processes might contribute to maintaining mutualistic microorganisms whilst keeping pathogens in check (Hooper et al., 2012). It seems likely that the immune system of high-ranking females better limits parasitic infections than those of low-ranking females. Their higher contact rate with other clan members (East et al., 2001) and monopolization of parasite-infected social resting sites and parts of carcasses (on which they feed) can then enable high-ranking females to absorb, establish and maintain a more diverse eukaryome.

To alleviate primer bias and handling problems arising from primers targeting mainly food items we used a multi-amplicon sequencing approach (Heitlinger, 2017). To assess to what extent these methods provide a quantitative estimate of the abundance at a particular level of resolution achieved between taxa, we correlated FEC or FOC with sequence reads. Our results indicate that sequencing based estimates were more sensitive both in the detection and the identification of parasite taxa than morphologically based FEC or FOC. We also found that the number of sequence reads was moderately to strongly positively correlated with FEC or FOC counts (Figure 2), which generally indicates that a degree of quantification was possible using amplicon sequencing, as reported by previous studies (Kartzinel et al., 2015; Pornon et al., 2016). For analyses of this kind, it is important to take into account the fact that taxa vary in the amount of DNA present in traditionally counted entities such as eggs, oocysts or whole individuals as this will influence the number of sequencing reads obtained (Blanckenhorn et al., 2016). The stronger correlations between the number of annotated sequence reads and coccidian FOC counts than between annotated sequence reads and helminth FEC may be due to the different life cycles of these parasites. Coccidian parasites live in the cells of the epithelial lining, thus contribute DNA via oocytes shed into the lumen of the intestines whereas adult helminths reside in the lumen of the intestines, thus potentially can contributed DNA both in the eggs they shed and from adult worms. The extent of discrepancies introduced by adult worm DNA would then depend on the relationship between egg numbers and helminth tissue in the DNA preparation. For some helminth parasites, this relationship may also depend on adult female/male ratios and other biological processes such as density-dependent effects on worm fecundity, which introduce biases in the estimate of hookworm burdens (Anderson and Schad, 1985).

Cestodes in the family Diphyllobothriidae produce eggs with a very similar morphology, thus reliable identification of *Spirometra* spp. and *Diphyllobothrium* spp. based on egg morphology is challenging (Thanchomnang et al., 2016). Our sequencing based taxonomic assignments suggest that eggs from both *Spirometra* spp. and *Diphyllobothrium* spp. were included in these egg counts. Similarly, *Ancylostoma* spp. have a typical strongyle egg type, hence differentiation between *Ancylostoma* spp. eggs and those of species such as *Haemonchus* spp. is difficult. Although *Haemonchus* spp. are parasites of ungulates, they might be detected in hyena feces by both amplicon sequencing and FEC after ingestion if hyenas fed on the viscera of an infected ungulate prey. The detection of *Haemonchus* spp. eggs in the feces of hyena does not indicate to us at present that these parasite species are members of the hyena's eukaryome. Our sequencing results revealed that our FOC counts for the order Coccidia (phylum Apicomplexa) likely comprised oocysts from species in three genera. We thus conclude that our amplicon sequencing approach offered an improved resolution and sensitivity over traditional egg or oocyst identification techniques and that combining the results from both approaches can provide complementary information.

Currently, little is known about the gastrointestinal biome of most wildlife species in “natural” ecosystems. Nevertheless, components of Darwinian fitness correlated with intestinal biome features can provide insight into the function of associated organisms for their host. At a more basic level, research on a broad range of wild mammals is required to permit the correct functional role (parasite, commensal or mutualist) to be assigned to individual taxa present in intestinal biomes, and in the case of predatory species to correctly differentiate passaging material such as parasites of prey from true host gastrointestinal biome constituents. For the latter, high resolution amplicon markers (e.g., COI; Hebert et al., 2003) and analysis of correlations of the composition of the apparent intestinal biome and ingested food items might offer solutions. Annotation of taxa with functional roles additionally requires databases collating such information (Poelen et al., 2014). Whereas studies on humans and laboratory animals have revealed the importance of the bacterial microbiome in host nutrition, physiology and immune processes, little is known about the impact of intestinal eukaryote diversity and composition on any host. We propose that the assessment of intestinal biomes in free-ranging wildlife in the context of host fitness in terms of survival or reproductive success can help to identify beneficial and adverse community compositions for different demographic or social categories of host populations and distinguish those from dysbiosis. The hologenome concept of evolution proposes that evolution in complex organisms should, in addition to considering interactions between an individual's genome and its environment, also consider its interactions with the products and physiological processes arising from the combined genomes of the microorganisms it hosts (Zilber-Rosenberg and Rosenberg, 2008; but see Douglas and Werren, 2016). This has led some to suggest that, during the current period of rapid environmental change, the plasticity of the gastrointestinal microbiome may help some vertebrate populations adjust in an appropriate manner (Alberdi et al., 2016). We suggest that research on the roles of intestinal biomes for humans and wildlife should in addition encompass both unicellular and multicellular eukaryotes, including those traditionally thought of as parasites and the vast majority of organism so far unknown for their impact on hosts, before we can arrive at a balanced view of the benefits and costs of different community compositions of intestinal biomes.

## Data deposition

Raw data has been deposited under accession number PRJNA386767 at NCBI Short Read Archive (SRA).

## Ethics statement

All protocols were non-invasive and adhered to the laws and guidelines of Tanzania. Permission to conduct research in Tanzania was granted to HH, ME, and SF by the Tanzania Commission for Science and Technology. Permission to undertake research within the Serengeti National Park was granted by the Tanzanian National Parks Authority, and the research was approved by the Tanzanian Wildlife Research Institute. The research was also approved by the Committee for Ethics and Animal Welfare of the Leibniz Institute for Zoo and Wildlife Research under the approval number 2008-11-02.

## Author contributions

EH, SF, HH, and ME designed the study, EH, SF, DT, HH, and ME collected the data, EH and SF analyzed the data, EH, SF, HH, and ME wrote the manuscript. All authors approved the final version of the manuscript.

### Conflict of interest statement

The authors declare that the research was conducted in the absence of any commercial or financial relationships that could be construed as a potential conflict of interest.
